# Role of CdTe Interface Structure on CdS/CdTe Photovoltaic Device Performance

**DOI:** 10.3390/ma16206812

**Published:** 2023-10-23

**Authors:** Niva K. Jayswal, Dipendra Adhikari, Indra Subedi, Ambalanath Shan, Nikolas J. Podraza

**Affiliations:** Department of Physics and Astronomy, Wright Center for Photovoltaics Innovation and Commercialization, The University of Toledo, Toledo, OH 43606, USA; niva.jayswal@rockets.utoledo.edu (N.K.J.); dipendra.adhikari@rockets.utoledo.edu (D.A.); indra.subedi@rockets.utoledo.edu (I.S.);

**Keywords:** glancing angle deposition (GLAD), thin film solar cell, GLAD CdTe interlayer, interface tailoring

## Abstract

Glancing angle deposition (GLAD) of CdTe can produce a cubic, hexagonal, or mixed phase crystal structure depending upon the oblique deposition angles (Φ) and substrate temperature. GLAD CdTe films are prepared at different Φ at room temperature (RT) and a high temperature (HT) of 250 °C and used as interlayers between the n-type hexagonal CdS window layer and the p-type cubic CdTe absorber layer to investigate the role of interfacial tailoring at the CdS/CdTe heterojunction in photovoltaic (PV) device performance. The Φ = 80° RT GLAD CdTe interlayer and CdS both have the hexagonal structure, which reduces lattice mismatch at the CdS/CdTe interface and improves electronic quality at the heterojunction for device performance optimization. The device performance of HT CdS/CdTe solar cells with Φ = 80° RT with 50 to 350 nm thick GLAD CdTe interlayers is evaluated in which a 250 nm interlayer device shows the best device performance with a 0.53 V increase in open-circuit voltage and fill-factor product and a 0.73% increase in absolute efficiency compared to the HT baseline PV device without an interlayer.

## 1. Introduction

Cadmium telluride (CdTe) is a photovoltaic (PV) absorber layer material with an absorption coefficient > 10^5^ cm^−1^ in the visible spectral range and a bandgap of ~1.5 eV which is well suited for single-junction PV devices [1,2,3,4,5,6,7]. CdTe-based PV devices have achieved 22.1% efficiency and have captured ~5% of the current world PV market [8]. CdTe can be deposited using different deposition methods such as close-space sublimation [9,10], electrochemical deposition [11], close-space vapor transport deposition [12,13], and sputtering [2,3,4,5,6]. Sputtered CdTe solar cells are widely studied in laboratory research as they can be fabricated at comparatively low temperatures (<250 °C), whereas close-space sublimation and vapor transport deposition require high processing temperatures (>500 °C) for the best CdTe cells, while electrochemical deposition requires acidic or basic media. Previously, all sputtered CdS/CdTe heterojunction solar cells with up to 14% efficiency have been reported [6]. The efficiency of CdTe solar cell devices can be increased by improving the conductivity of the front contact with novel transparent conducting oxides with higher conductivity and low resistance. Other potential measures include barrier-free back contacts and fewer defects at the CdS/CdTe interface by reducing the high lattice mismatch between the n-type hexagonal CdS window layer and the p-type cubic CdTe absorber layer [14,15]. The lattice mismatch between hexagonal wurtzite-structured CdS and cubic zinc blende CdTe can be reduced by optimizing the interdiffusion of CdS and CdTe at the CdS/CdTe interface or controlling the microstructure of CdTe at the interface to improve the electronic quality [16,17,18]. The optimized interdiffusion of CdS and CdTe at the interface reduces the lattice mismatch due to the decreased interatomic distance by the substitution of lower atomic radii sulfur onto tellurium sites. The crystallite orientation and size can be controlled by manipulating the microstructure at the interface, which can be used to reduce the lattice mismatch at the CdS/CdTe heterojunction interface to improve the structural and electronic quality, leading to better PV device performance.

Glancing angle deposition (GLAD) can be employed to produce polycrystalline thin films with different microstructures for use at the CdS/CdTe interface. In GLAD, the substrate normal and target normal are at an oblique source flux angle (Φ). Microstructural control of the CdTe films by oblique angle deposition can engineer different crystal structures and crystallite orientations of the polycrystalline thin films. In previous studies, an X-ray diffraction (XRD) analysis of room temperature (RT) GLAD CdTe has been shown to exhibit cubic [19,20,21,22], mixed phase [20], or hexagonal CdTe crystal structures for the films prepared by varying source flux angles from Φ = 0° to 80° using different physical vapor deposition techniques, such as thermal evaporation and sputtering [23,24,25,26]. The presence of overlapping cubic and hexagonal phase diffraction peaks implies the mixed phase, with the dominant peaks being the cubic phase.

The metastable hexagonal wurtzite phase of CdTe is produced when sputtered at an oblique angle (Φ = 80°) deposition at RT, as opposed to the more stable cubic zinc blende CdTe [23]. However, XRD patterns of 250 °C GLAD CdTe films exhibit only a cubic or mixed phase structure at any oblique source flux angle [26]. The Φ = 40° GLAD CdTe thin film fabricated at 250 °C shows the highest preferential crystallite orientation and has the largest crystallite size among the high-temperature (HT) GLAD polycrystalline CdTe films [26]. More preferential crystallite orientations and the strong preference for the formation of the stable cubic phase over the metastable wurtzite phase occur at high-temperature CdTe in general due to additional thermal energy increasing the diffusion length of precursors on the surface and facilitating the formation of the more thermodynamically stable crystal structure compared to the material deposited at room temperature. The largest crystallite size and most preferential orientation are observed for high-temperature GLAD CdTe prepared at Φ = 40°, possibly as an effect of a slightly lower nucleation density. Hexagonal and mixed phase CdTe produced by higher deposition angles exhibit a crystal structure more comparable with hexagonal CdS than the zinc blende cubic phase of CdTe. When applied at the CdS/CdTe interface, PV device performance can be optimized by reducing lattice mismatch at the n/p heterojunction interface. Previously, an RT-deposited CdTe solar cell with an RT GLAD CdTe interlayer demonstrated a 0.9% increase in absolute efficiency [23].

This work investigates the role of interfacial tailoring of the phase structure and the resulting effect on the HT CdS/CdTe solar cell device performance. GLAD is used to produce interlayers with wurtzite and mixed phase crystal structures as well as strongly preferentially oriented cubic CdTe, which are then applied between the thin (~100 nm) wurtzite hexagonal CdS window layer and thicker (~2000 nm) zinc blende cubic CdTe absorber. GLAD interlayer deposition conditions are chosen to modify the phase structure (hexagonal and mixed phase), degree of preferential crystallite orientation, and crystallite size. As a result, the impacts of tailoring the phase structure and crystallite orientation at the CdS/CdTe heterojunction interface on device performance are studied using interlayers prepared at Φ = 80° RT with the hexagonal structure and at Φ = 40° HT with the largest crystallite size and most preferentially oriented cubic phase crystallites.

Here, we have incorporated 50 to 350 nm thick Φ = 80° RT GLAD CdTe interlayers and a 150 nm thick Φ = 40° HT GLAD interlayer in CdS/CdTe PV devices with the remainder of the bulk CdTe fabricated at 250 °C, and we have compared the device performance with the baseline PV devices without interlayers fabricated at 250 °C and RT. The hexagonal phase GLAD CdTe interlayers adjacent to the hexagonal CdS heterojunction partner reduce the lattice mismatch between it and cubic CdTe. This enables better tailoring of the phase structure resulting in improved electronic quality at the interface and is expected to enhance PV device performance. PV device performance parameters are determined for all the devices discussed in this work in terms of efficiency, open-circuit voltage (*V_oc_*), short-circuit current density (*J_sc_*), fill-factor (*FF*), and *V_oc_* × *FF* product. The ideality factors of baseline devices, the best performance device, and the Φ = 40° HT GLAD interlayer device are determined to evaluate the diode behavior of these devices. The PV device efficiency, *V_oc_*, and *FF* have been improved and are the highest with the incorporation of a 250 nm Φ = 80° RT GLAD CdTe interlayer between CdS and bulk CdTe layers of an otherwise standard HT CdS/CdTe solar cell due to the reduced lattice mismatch and strain at the interface. Also, the important device parameter, the *V_oc_* × *FF* product, is the highest for the 250 nm Φ = 80° RT GLAD CdTe interlayer device due to better tailoring of the phase structure improving the electronic quality of the interface. An optimized morphology of the GLAD CdTe interface has been identified to improve the PV device performance due to an improved n/p heterojunction interface.

## 2. Materials and Methods

### 2.1. Standard CdS/CdTe Solar Cell Fabrication

**Figure 1:**
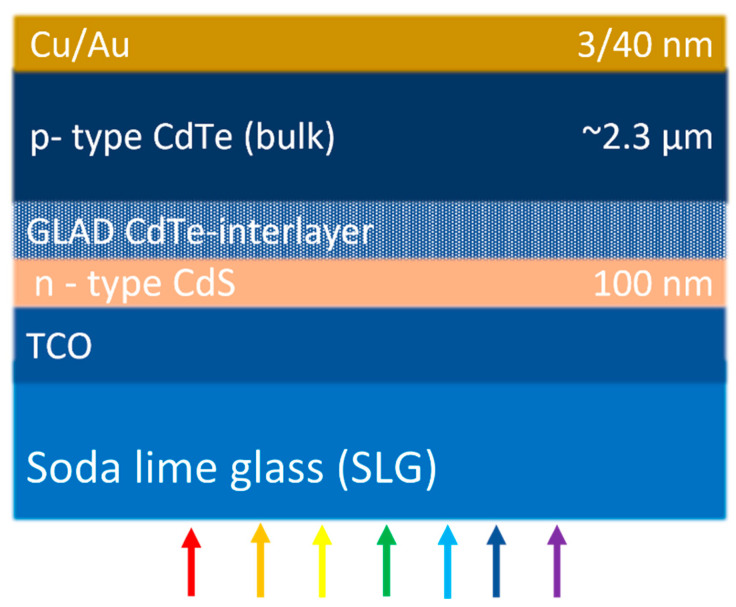
CdS/CdTe solar cell structure with a thin GLAD CdTe (hexagonal, cubic, or mixed phase) interlayer between hexagonal wurtzite CdS and cubic zinc blende CdTe bulk layer.

Standard CdS/CdTe heterojunction solar cells have been fabricated with and without GLAD CdTe interlayers in the superstrate configuration (TEC-12D/CdS/GLAD CdTe-interlayer/CdTe/Cu/Au) as shown in Figure 1. TEC-12D (NSG Pilkington North America, Northwood, OH, USA) [23] is a commercial soda lime glass coated with 300 to 400 nm fluorine-doped tin oxide (SnO_2_:F) and a 50 nm high-resistivity transparent (HRT) SnO_2_ layer on top of SnO_2_:F. Prior to the CdS/CdTe solar cell fabrication, the TEC-12D glass superstrate is ultrasonically cleaned in two steps with and without detergent for 45 min, and then rinsed with deionized water followed by drying with nitrogen gas. The 100 nm n-type CdS window layer is prepared on TEC-12D followed by p-type CdTe absorber layer deposition using RF magnetron sputtering at normal incidence to fabricate all CdS/CdTe cells, with or without a GLAD CdTe interlayer. For standard baseline CdS/CdTe solar cells without interlayers, a 2.2 to 2.5 μm thick bulk CdTe is deposited at HT and RT following CdS deposition, but for cells with an interlayer, the bulk CdTe is deposited after the GLAD CdTe interlayer. After the application of the CdTe bulk layer, CdCl_2_ treatment is carried out for 30 min in dry air at 387 °C, followed by a methanol wash and nitrogen drying as has been previously optimized [2,23,27,28,29]. To complete the solar cell, a back contact of 3 nm Cu and 40 nm Au is deposited at RT using thermal evaporation onto the CdCl_2_-treated CdS/CdTe stack with a dot shadow mask. A 4″ × 4″ superstrate is used to fabricate 144 individual dot solar cells, each with an area of 0.126 cm^2^.

To increase the p-type conductivity of the CdTe absorber layer, Cu diffusion is performed by annealing the complete solar cell device at 150 °C for 30 min in air, enabling the Cu to diffuse into bulk CdTe rather than remaining as a thin layer. The details of the deposition parameters for each layer of the solar cell are listed in Table 1.

### 2.2. Interlayer CdTe Deposition

**Figure 2:**
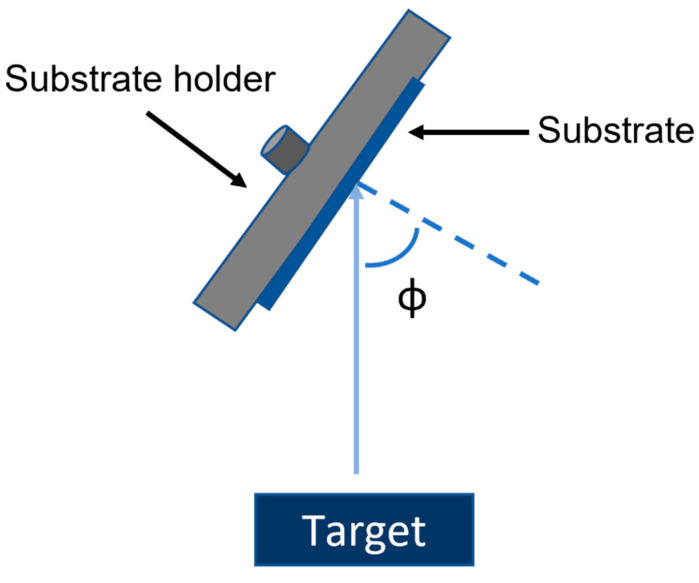
Schematic of glancing angle deposition (GLAD) sputtering at an oblique angle Φ.

GLAD CdTe thin films of different thicknesses are produced at different oblique angles (Φ = 80° and 40°) using RF magnetron sputtering with the schematic shown in Figure 2. Both the GLAD CdTe interlayer and bulk CdTe films are deposited using a 7.62 cm diameter CdTe target (99.999% pure, Kurt. J. Lesker Co., Jefferson Hills, PA, USA) in 15 mTorr Ar gas pressure at 100 W target power. At the normal incidence (Φ = 0°) deposition, the distance between the substrate and target centers is 13.4 cm. Previously, RT GLAD CdTe prepared at Φ = 80° has been shown to have a hexagonal wurtzite crystal phase [23]. Also, a 0.9% increase in absolute efficiency of the RT CdS/CdTe solar cell has been shown by incorporating such a film of 100 nm thickness as an interlayer compared to a standard baseline RT CdS/CdTe solar cell without an interlayer [23]. The hexagonal structure of Φ = 80° RT CdTe has been used as an interlayer due to its similar crystal structure to hexagonal CdS, reducing the lattice mismatch between hexagonal CdS and cubic bulk CdTe, with the resulting phase structure improving the electronic quality at the n/p heterojunction interface and increasing device performance. HT GLAD CdTe prepared at Φ = 40° has the highest preferential crystallite orientation and larger crystallite size relative to normal incidence cubic CdTe and is used to generate a more ordered material present at the heterojunction than the baseline. The Φ = 40° HT GLAD CdTe device has been studied as it has the greatest degree of preferential crystallite orientation and largest crystallite size among the GLAD films studied which reduce grain boundaries with the potential to reduce the photo-generated charge carrier recombination at the interface leading to improved device performance. Here, Φ = 80° RT GLAD CdTe interlayer films prepared under the same conditions with different thicknesses of 50, 100, 150, 200, 250, 300, and 350 nm and a 150 nm Φ = 40° HT GLAD CdTe interlayer have been incorporated between the wurtzite hexagonal n-type CdS window layer and zinc blende cubic p-type CdTe bulk absorber prepared at HT. The 50 to 350 nm thick Φ = 80° RT GLAD CdTe films are expected to have a hexagonal crystal structure, as was indicated previously for >1000 nm films made under the same conditions before and after the CdCl_2_ treatment [23]. The Φ = 40° HT GLAD CdTe > 1000 nm [26] has a predominately cubic mixed phase structure with preferential orientation of H(002)/C(111) and C(222) crystallites; therefore, a 150 nm GLAD CdTe film is deposited in the same manner. The structural properties of CdTe films can tune the optoelectronic properties of PV device optimization resulting from the GLAD CdTe tailoring of the material structure between hexagonal CdS and cubic CdTe at the interface. The deposition rate of the target material at the center of the substrate is ~31 nm/min for Φ = 0° HT CdTe, ~15 nm/min for Φ = 80° RT GLAD CdTe, and ~28 nm/min for Φ = 40° HT GLAD CdTe. For the PV device characterization, the current density versus voltage (*J-V*) has been measured using a Keithley 2440 digital source meter in the dark and under one sun AM1.5 (100 mW/cm^2^) illumination with a 450 W Xenon light source (Oriel, Model 9119, Newport, CA, USA) [15,23,25,27,29].

## 3. Results and Discussion

Standard CdTe devices without GLAD CdTe interlayers are fabricated with the bulk CdTe deposited at HT and RT and having the device structure shown in Figure 1. HT bulk CdTe films have pronounced crystallinity and more preferentially orientated crystallites with larger grain sizes when compared to RT bulk CdTe films. The larger grains of HT bulk CdTe reduce grain boundaries and defect state density, leading to reduced photo-generated carrier recombination. As expected, the device efficiency of the standard HT CdTe cell without the interlayer is higher than the standard RT CdTe cell without an interlayer. *J_sc_*, *V_oc_*, and *FF* increase from 21.36 mAcm^−2^, 0.767 V, and 58.62% for the RT baseline device to 22.15 mAcm^−2^, 0.825 V, and 64.22% for the HT baseline device. The standard HT CdTe device shows a 3.2% absolute increase in efficiency when compared to the standard RT CdTe device, increasing from 9.59 to 12.76%.

Building from the baseline device structure, CdS/CdTe solar cells have been fabricated incorporating Φ = 80° RT GLAD CdTe interlayers with different thicknesses from 50 to 350 nm and a Φ = 40° HT GLAD CdTe interlayer for comparison with devices incorporating bulk CdTe deposited at 250 °C without interlayers. To investigate whether a hexagonal crystal structure interlayer may improve the performance of typical HT CdS/CdTe solar cells by minimizing strain and lattice mismatch, the Φ = 80° RT GLAD hexagonal structured CdT interlayer of different thicknesses, dn, (n = 1 to 7) = 50, 100, 150, 200, 250, 300, and 350 nm is incorporated between the wurtzite hexagonal CdS n-type heterojunction partner and zinc blende cubic CdTe p-type absorber. Previously, it had been observed that the hexagonal structured RT GLAD CdTe interlayer reduces the lattice mismatch between hexagonal CdS and cubic bulk CdTe, reducing the defect density and carrier recombination at the heterojunction n/p interface in devices with RT CdTe bulk absorbers and improving the device performance by a 0.9% absolute increase in efficiency [23]. The potentially mixed phase but predominately cubic HT GLAD CdTe interlayer prepared at Φ = 40°, having the largest crystallite size and highest preferential crystallite orientation with only H(002)/C(111) and C(222) diffraction peaks [26], is incorporated in standard HT CdS/CdTe solar cells to investigate the impact of the reduced grain boundaries and preferential crystallite orientation of the HT GLAD interlayer on PV device performance.

**Figure 3:**
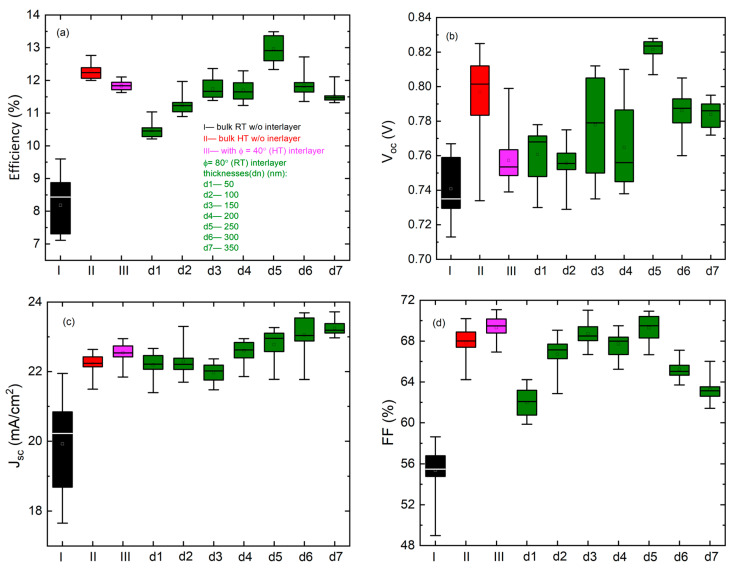
(**a**) Efficiency, (**b**) open-circuit voltage (*V_oc_*), (**c**) short-circuit current density (*J_sc_*), and (**d**) fill-factor (*FF*) for the 20 best CdTe photovoltaic devices with and without GLAD CdTe interlayers prepared with varying temperatures, source flux angles, and thicknesses.

**Figure 4:**
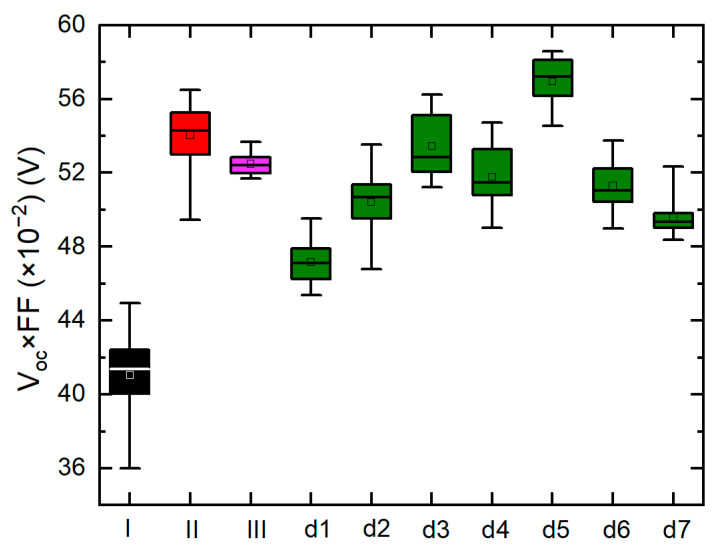
The product of *V_oc_* and *FF* for the 20 best (highest efficiency) CdTe photovoltaic devices with and without GLAD CdTe interlayers prepared with varying temperatures, source flux angles, and thicknesses.

Figure 3 and Figure 4 show the statistical distribution (best 20 performing cells) of device efficiency, *V_oc_*, *J_sc_*, *FF*, and the product of *V_oc_* and *FF* (*V_oc_* × *FF*) for the different device configurations tested and comparison of device performance parameters are shown in Table 2. These devices are fabricated with varying deposition parameters as those (I, II) without interlayers fabricated at RT and 250 °C as baselines; (III) with a 250 °C deposited bulk absorber and a Φ = 40° mixed phase interlayer also deposited at a high temperature, which exhibits highly preferential orientation; and (dn, n = 1–7) with 250 °C deposited bulk absorbers and different Φ = 80° RT interlayers of different thickness which exhibit a hexagonal crystal structure. The device with a 250 nm Φ = 80° RT GLAD CdTe interlayer shows the highest efficiency and *V_oc_* × *FF* increasing from 12.76 to 13.49% (absolute 0.73% increase) and 0.53 to 0.58 V (9.4% increase), respectively, when compared to the best performing standard HT baseline device. The *V_oc_* × *FF* product is the key factor for voltage at the maximum power point as the solar cells are designed to be operated at the maximum power point in commercial use [30]. Practically, this also enables a more direct comparison of device performance when subtle differences in absorber layer thickness impact *J_sc_* and the resulting device efficiency, while *V_oc_* and *FF* may be less strongly impacted. Previous studies have shown that some portion of metastable hexagonal CdTe remains after the higher temperature CdCl_2_ process, so these improvements in device performance may be attributed to a similar effect whereby hexagonal phase crystallites are present in sufficient quantities to reduce lattice mismatch at the CdS/CdTe interface, but not to the extent that the electronic performance of the bulk layer is reduced. Reducing the lattice mismatch improves the n/p interface, making it less defective with a higher electronic quality which also reduces carrier recombination and increases the device performance [31,32].

When comparing the device performance with different thicknesses of this interlayer, the average *J_sc_* initially decreases and then increases with the increasing interlayer thickness. The initial decrease in *J_sc_* with small interlayer thicknesses can be attributed to the recombination of the photo-generated charge carriers in the bulk CdTe absorber. Although recombination may also occur in these thin GLAD interlayers, devices with thicker GLAD interlayers show increased *J_sc_*, implying that the electronic quality of the bulk CdTe may be compromised when deposited upon thin GLAD interlayers. The latter increase in *J_sc_* with increasing interlayer thickness can be attributed to the increased total CdTe thickness of the bulk and the GLAD interlayer, which consistently increases by ~0.2 mA/cm^2^ per each 50 nm increase in the interlayer and total thickness. Previously, it has been shown that the *J_sc_* of CdS/CdTe solar cells increases with increasing CdTe thickness up to ~3 or 4 μm [33,34,35,36]. The total thickness of the CdTe absorber, consisting of both the bulk and GLAD interlayer, in any of these devices does not exceed 4 μm, which may have led to a slight increase in *J_sc_* when the interlayer is incorporated to increase the total absorber layer thickness.

The device with a 250 nm Φ = 80° RT GLAD CdTe interlayer device shows the highest *V_oc_* due to a reduction in the lattice mismatch from tailoring the phase structure at the interface. The *FF*s of all interlayer devices except the device with the 250 nm interlayer are comparable to or less than the *FF*s of HT baseline devices, which may be due to the presence of parasitic resistance leading to reduced diode characteristics of the solar cell. The reduced *V_oc_* and *FF* for devices with GLAD interlayers greater than or less than 250 nm show that interface modification is not optimized. The device incorporating the 250 nm GLAD interlayer shows the highest *FF* among all devices, which can be attributed to lower parasitic resistive losses. The optimization observed when incorporating a 250 nm thick Φ = 80° RT GLAD CdTe interlayer compared to thinner interlayers may be due to a decreasing strain gradient in the GLAD CdTe with increasing thickness, and it is the highest when in contact with CdS [23,37,38]. However, structural defects also increase with the interlayer thickness due to the relatively low density and pronounced columnar structure of the GLAD interlayer compared to bulk CdTe upon which bulk CdTe grows. Here, a device incorporating a 250 nm GLAD interlayer may be optimized by the reduction of strain at the interlayer/bulk CdTe interface with an interlayer that is not sufficiently thick to negatively impact the subsequent growth of bulk CdTe.

Implementing the Φ = 40° HT GLAD CdTe interlayer in a standard HT CdTe device does not show improvement in device performance. In previous work, the XRD patterns of Φ = 40° HT GLAD CdTe did not exhibit diffraction peaks that can be attributed solely to the hexagonal phase. Predominant diffraction peaks are C(222) for Φ = 40° HT GLAD CdTe and C(400) and C(331) for Φ =0° HT CdTe. The lattice mismatch between the crystal planes of C(222)-oriented crystallites found predominately in Φ = 40° HT GLAD CdTe and hexagonal CdS might be higher than that between those associated with C(400) or C(331)-oriented crystallites found in the more randomly oriented Φ = 0° of HT CdTe. Hence, a larger lattice mismatch between the Φ = 40° HT GLAD CdTe interlayer and CdS than the HT CdTe and CdS causes an increase in carrier recombination at that interface due to the presence of increased strain, unpassivated bonds, or both, and a decrease in *V_oc_*, *V_oc_* × *FF*, and PV device efficiency for the Φ = 40° HT GLAD CdTe interlayer PV device compared to the HT baseline device. *J_sc_* of the Φ = 40° HT GLAD CdTe interlayer is higher than that of the HT baseline device, which can be attributed to the greater sum of the bulk and GLAD CdTe interlayer leading to increased photo-generation and collection of carriers. The FF of the Φ = 40° HT GLAD CdTe interlayer is higher than the HT baseline device due to improved diode characteristics, as observed from an improved ideality factor. These results indicate that the most preferentially oriented HT GLAD CdTe interlayer is not beneficial to improving the PV device performance, primarily by its negative effect on *V_oc_*. Based on this observation, varying the Φ = 40° HT GLAD CdTe interlayer thickness would not be expected to optimize the device; however, a complete series, as for those based on the Φ = 80° RT GLAD CdTe interlayer, has not be produced. For the Φ = 40° HT GLAD CdTe interlayer device, the highest strain and lattice mismatch occurs at the CdS/GLAD interlayer interface. The greater level of preferential orientation in this GLAD interlayer than in the baseline is likely responsible for the lower *V_oc_*. Although the strain will decrease with the increasing interlayer thickness, the structural defects occurring with the increasing GLAD interlayer thickness due to the relatively low density of the GLAD interlayer compared to bulk CdTe upon which bulk CdTe grows will reduce the *FF* [23,37,38].

**Figure 5:**
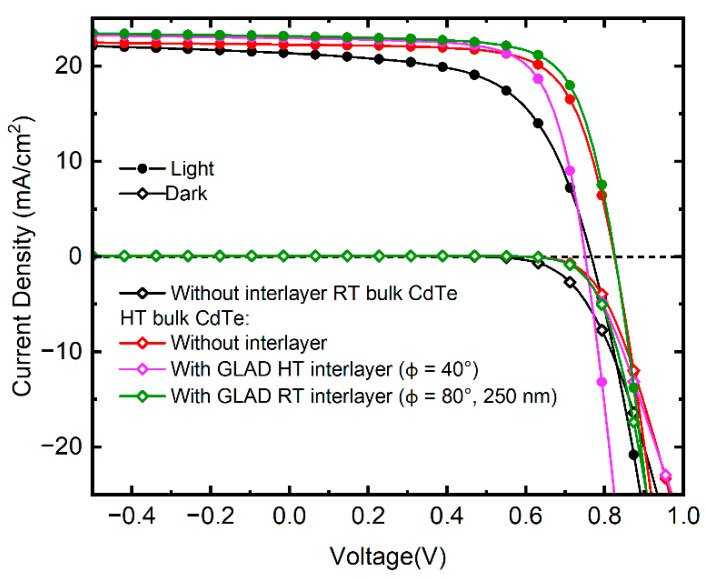
Dark and light current-voltage characteristics of CdTe solar cells with GLAD HT interlayer (Φ = 40° and 80°) and without (RT and HT bulk CdTe) GLAD CdTe interlayers.

**Figure 6:**
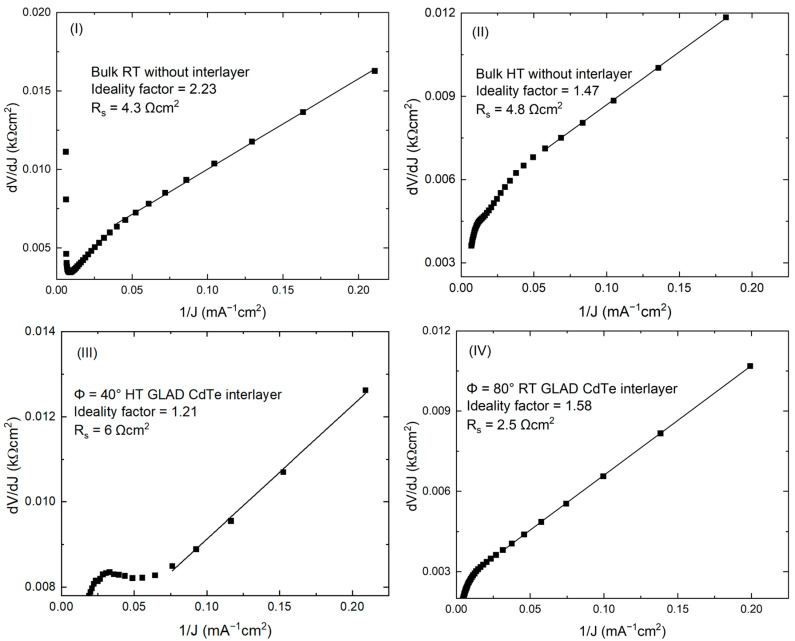
Dark *J*-*V* characteristic for the best performing CdTe solar cells plotted as d*V*/d*J* as a function of 1/*J* to determine ideality factors and series resistances: (**I**) bulk RT CdTe device without an interlayer, (**II**) bulk HT CdTe device without an interlayer, (**III**) Φ = 40° HT GLAD interlayer device, and (**IV**) Φ = 80° RT 250 nm GLAD interlayer device.

Figure 5 shows the light and dark *J-V* characteristics of the highest efficiency RT and HT baseline PV devices without interlayers and Φ = 40° HT and 250 nm Φ = 80° RT GLAD CdTe interlayer devices. The *J-V* behavior of the solar is described by the diode equation [39,40,41]:

(1) $$J=J_{0}\exp\left[\frac{q}{Ak_{B}T}\left(V-JR_{S}\right)\right]+G_{SH}V-J_{L},$$

where *J*_0_ is the reverse saturation current, *A* is the diode ideality factor, *q* is the electronic charge, *k_B_* is Boltzmann’s constant, *T* is the temperature, *R_s_* is series resistance, *G_SH_* is shunt conductance, and *J_L_* is the load current density = *J_sc_* = 0 for dark conditions. From the experimental dark *J-V*, the solar cell diode ideality factors and series resistances are determined from the behavior of d*V*/d*J* as a function of 1/*J* as shown in Figure 6. The slope and the intercept of the linear fit of d*V*/d*J* as a function of 1/*J* are used to find the ideality factor and series resistance, respectively. The series resistances are found to be 4.3 and 4.7 Ωcm^2^ for the RT and HT baseline devices with ideality factors of 2.2 and 1.5, respectively. Similarly, the series resistances are 6.0 and 2.7 Ωcm^2^ for the Φ = 40° HT and 250 nm Φ = 80° RT GLAD CdTe interlayer devices with ideality factors of 1.2 and 1.5, respectively. Similar values of *A* and *R_s_* had been reported previously for these types of device structures [23,39,41,42]. The lower values of *A* and *R_s_* for the HT CdTe baseline device compared to the RT CdTe baseline device clearly indicate improvement in the diode performance parameters and increased solar cell device performance parameters due to larger crystallite size and reduced grain boundaries, lowering the defect density and reducing the charge carrier recombination in the HT bulk CdTe cell. The almost equal ideality factor and series resistance values for the Φ = 80° RT GLAD CdTe interlayer device compared to the HT baseline device indicate the enhancement in the diode performance parameter due to the improved n/p heterojunction interface, and the improved electronic quality of the n/p interface leading to the increase in *V_oc_*, *FF*, and the overall device performance parameters [30,33,36]. Comparable values for the Φ = 40° HT interlayer device with the HT baseline device indicate the similar diode performance parameters and similar device performance.

## 4. Conclusions

CdS/CdTe PV devices are fabricated incorporating GLAD interlayers with varying deposition parameters such as temperature, source flux angle, and interlayer thicknesses. Device performance is compared in terms of efficiency, *V_oc_*, *J_sc_*, *FF*, and the product of *V_oc_* and *FF*. RT and 250 °C standard CdS/CdTe solar cells are fabricated without the GLAD CdTe interlayer as the baseline PV devices. The HT standard CdTe PV device incorporating a 250 nm Φ = 80° RT GLAD hexagonal CdTe interlayer shows a 0.73% absolute increase in efficiency when compared to the HT baseline CdTe solar cell without an interlayer. This optimization may be due to a reduction of strain at the interlayer / bulk CdTe interface without negatively impacting the growth of bulk CdTe due to a structural defect from the columnar structure of the underlying GLAD CdTe. This device performance indicates the presence of some hexagonal CdTe to reduce the lattice mismatch at the interface with CdS without reducing the electronic performance of the bulk CdTe absorber layer. Moreover, a ~0.53 V increase in *V_oc_* × *FF* has been found when comparing the 250 nm interlayer device to the HT CdTe baseline solar cell, which could be attributed to better interfacial tailoring at the CdS/CdTe heterojunction interface in the PV device.

## Figures and Tables

**Table 1:** Deposition parameters for CdS/CdTe solar cell component. RT indicates room temperature.

Layer	Deposition Technique	Temperature	Thickness (nm)	Deposition Parameters			
				Φ (°)	Target Power (W)	Pressure (mTorr)	Ar Flow (sccm)
CdS	RF sputtering	250 °C	100		200	15	23
GLAD CdTe interlayer	RF sputtering	RT	50–350	80	100	15	23
		250 °C	150	40	100	15	23
Bulk CdTe	RF sputtering	RT	2.2–2.4 μm	0	100	15	23
		250 °C	2.2–2.4 μm	0	100	15	23
Cu	Thermal evaporation	RT	3	-	-	10^−3^	-
Au	Thermal evaporation	RT	40	-	-	10^−3^	-

**Table 2:** Comparison of device performance parameters of 20 highest efficiency CdTe solar cells with and without GLAD interlayers prepared as functions of deposition temperature, source flux angle (Φ), and interlayer thicknesses.

CdTe Deposition					Device Parameters				Best Cell
Bulk CdTe Temperature	Interlayer CdTe Temperature	Φ	Interlayer Thickness (nm)		*V_oc_* (V)	*J_sc_* (mAcm^−2^)	*FF* (%)	Efficiency (%)	*V_oc_* × *FF* (V) (× 10^−2^)
RT	w/o interlayer	-	-	Best cell	0.767	21.36	58.62	9.59	44.96
				Average of 20 cells	0.741 ± 0.017	19.9 ± 1.4	55.4 ± 2.3	8.2 ± 0.8	
HT	w/o interlayer	-	-	Best cell	0.825	22.15	64.22	12.76	52.98
				Average of 20 cells	0.797 ± 0.022	22.2 ± 0.3	67.8 ± 1.6	12.3 ± 0.2	
	HT	40°	150	Best cell	0.751	22.95	70.25	12.11	52.76
				Average of 20 cells	0.757 ± 0.015	22.5 ± 0.3	69.3 ± 1.1	11.8 ± 0.1	
	RT	80°	50	Best cell	0.778	22.28	63.66	11.04	49.53
				Average of 20 cells	0.761 ± 0.015	22.1 ± 0.3	62.0 ± 1.4	10.5 ± 0.2	
		80°	100	Best cell	0.783	22.22	68.65	11.94	53.75
				Average of 20 cells	0.756 ± 0.012	22.3 ± 0.4	66.7 ± 1.6	11.2 ± 0.3	
		80°	150	Best cell	0.808	22.06	69.37	12.36	56.05
				Average of 20 cells	0.778 ± 0.028	21.9 ± 0.3	68.7 ± 1.3	11.7 ± 0.3	
		80°	200	Best cell	0.801	22.46	68.33	12.29	54.73
				Average of 20 cells	0.765 ± 0.025	22.6 ± 0.3	67.7 ± 1.2	11.7 ± 0.3	
		80°	250	Best cell	0.826	23.15	70.54	13.49	58.26
				Average of 20 cells	0.821 ± 0.005	22.77 ± 0.4	69.29 ±1.3	12.9 ± 0.4	
		80°	300	Best cell	0.800	23.69	67.10	12.717	53.68
				Average of 20 cells	0.786 ± 0.011	23.0 ± 0.5	65.2 ± 1.0	11.8 ± 0.3	
		80°	350	Best cell	0.793	23.13	66.01	12.11	52.35
				Average of 20 cells	0.784 ± 0.007	23.2 ± 0.2	63.2 ± 1.2	11.5 ± 0.2	

## Data Availability

The data presented in this study are available on request from the corresponding author.

## References

[1] Mitchell K., Fahrenbruch A.L., Bube R.H. (1977). Photovoltaic determination of optical-absorption coefficient in CdTe. J. Appl. Phys..

[2] Gupta A., Parikh V., Compaan A.D. (2006). High efficiency ultra-thin sputtered CdTe solar Cells. Sol. Energy Mater. Sol. Cells.

[3] Koirala P., Li J., Yoon H.P., Aryal P., Marsillac S., Rockett A.A., Podraza N.J., Collins R.W. (2016). Through-the-glass spectroscopic ellipsometry for analysis of CdTe thin-film solar cells in the superstrate configuration. Prog. Photovolt. Res. Appl..

[4] Morales-Acevedo A. (2006). Thin film CdS/CdTe solar cells: Research perspectives. Sol. Energy.

[5] Paudel N., Wieland K., Compaan A. (2012). Ultrathin CdS/CdTe solar cells by sputtering. Sol. Energy Mater. Sol. Cells.

[6] Gupta A., Compaan A.D. (2004). All-sputtered 14% CdS/CdTe thin-film solar cell with ZnO: Al transparent conducting oxide. Appl. Phys. Lett..

[7] Wu X. (2004). High-efficiency polycrystalline CdTe thin-film solar Cells. Sol. Energy..

[8] Green M., Dunlop E., Hohl-Ebinger J., Yoshita M., Kopidakis N., Hao X. (2021). Solar cell efficiency tables (Version 57). Prog. Photovolt. Res. Appl..

[9] Schaffner J., Motzko M., Tueschen A., Swirschuk A., Schimper H.-J., Klein A., Modes T., Zywitzki O., Jaegermann W. (2011). 12% efficient CdTe/CdS thin film solar cells deposited by low-temperature close space sublimation. J. Appl. Phys..

[10] Britt J., Ferekides C. (1993). Thin-film CdS/CdTe solar cell with 15.8% efficiency. Appl. Phys. Lett..

[11] Savadogo O. (1998). Chemically and electrochemically deposited thin films for solar energy materials. Sol. Energy Mater. Sol. Cells.

[12] Vigil-Galán O., Vaillant L., Mendoza-Pérez R., Contreras-Puente G., Vidal-Larramendi J., Morales-Acevedo A. (2001). Influence of the growth conditions and postdeposition treatments upon the grain boundary barrier height of CdTe thin films deposited by close space vapor transport. J. Appl. Phys..

[13] Mendoza-Pérez R., Sastre-Hernández J., Contreras-Puente G., Vigil-Galán O. (2009). CdTe solar cell degradation studies with the use of CdS as the window material. Sol. Energy Mater. Sol. Cells.

[14] Rioux D., Niles D.W., Höchst H. (1993). ZnTe: A potential interlayer to form low resistance back contacts in CdS/CdTe solar Cells. J. Appl. Phys..

[15] Subedi K.K., Bastola E., Subedi I., Song Z., Bhandari K.P., Phillips A.B., Podraza N.J., Heben M.J., Ellingson R.J. (2018). Nanocomposite (CuS)_x_(ZnS)_1−x_ thin film back contact for CdTe solar cells: Toward a bifacial device. Sol. Energy Mater. Sol. Cells.

[16] Nakamura K., Gotoh M., Fujihara T., Toyama T., Okamoto H. (2003). Influence of CdS window layer on 2-μm thick CdS/CdTe thin film solar Cells. Sol. Energy Mater. Sol. Cells.

[17] Dobson K.D., Visoly-Fisher I., Hodes G., Cahen D. (2000). Stability of CdTe/CdS thin-film solar Cells. Sol. Energy Mater. Sol. Cells.

[18] Chou H., Rohatgi A., Jokerst N., Kamra S., Stock S., Lowrie S., Ahrenkiel R., Levi D. (1996). Approach toward high efficiency CdTe/CdS heterojunction solar Cells. Mater. Chem. Phys..

[19] Gu P., Zhu X., Wu H., Yang D. (2018). Regulation of substrate-target distance on the microstructural, optical and electrical properties of CdTe films by magnetron sputtering. Materials.

[20] Kulkarni R., Rondiya S., Pawbake A., Waykar R., Jadhavar A., Jadkar V., Bhorde A., Date A., Pathan H., Jadkar S. (2017). Structural and optical properties of CdTe thin films deposited using RF magnetron sputtering. Energy Procedia.

[21] Hosseinpanahi F., Raoufi D., Ranjbarghanei K., Karimi B., Babaei R., Hasani E. (2015). Fractal features of CdTe thin films grown by RF magnetron sputtering. Appl. Surf. Sci..

[22] Hegedus S.S., Luque A. (2003). Status, trends, challenges and the bright future of solar electricity from photovoltaics. Handbook of Photovoltaic Science and Engineering.

[23] Adhikari D., Junda M.M., Bastola E., Koirala P., Ellingson R.J., Collins R.W., Podraza N.J. (2020). Glancing angle deposited CdTe: Nanostructured films and impact on solar cell performance. Surf. Coat. Technol..

[24] Ehsani M., Dizaji H.R., Azizi S., Mirmahalle S.G., Siyanaki F.H. (2013). Optical and structural properties of cadmium telluride films grown by glancing angle deposition. Phys. Scr..

[25] Adhikari D., Koirala P., Junda M.M., Collins R.W., Podraza N.J. Glancing angle deposited CdTe: Optical properties and structure. Proceedings of the IEEE 7th World Conference on Photovoltaic Energy Conversion (WCPEC) (A Joint Conference of 45th IEEE PVSC, 28th PVSEC & 34th EU PVSEC) IEEE.

[26] Jayswal N.K. Deposition Temperature Dependence of Optical and Structural Properties of Glancing Angle Deposited CdTe. Proceedings of the 64th Annual SVC Technical Conference.

[27] Paudel N.R., Young M., Roland P.J., Ellingson R.J., Yan Y., Compaan A.D. (2014). Post-deposition processing options for high-efficiency sputtered CdS/CdTe solar Cells. J. Appl. Phys..

[28] Compaan A.D., Gupta A., Lee S., Wang S., Drayton J. (2004). High efficiency, magnetron sputtered CdS/CdTe solar Cells. Sol. Energy.

[29] Tuteja M., Koirala P., Palekis V., MacLaren S., Ferekides C.S., Collins R.W., Rockett A.A. (2016). Direct observation of CdC_l2_ treatment induced grain boundary carrier depletion in CdTe solar cells using scanning probe microwave reflectivity based capacitance measurements. J. Phys. Chem. C..

[30] Liu Z., Siekmann J., Klingebiel B., Rau U., Kirchartz T. (2021). Interface optimization via fullerene blends enables open-circuit voltages of 1.35 V in CH_3_NH_3_Pb(I_0.8_Br_0.2_)_3_ solar Cells. Adv. Energy Mater..

[31] Song T., Kanevce A., Sites J.R. (2016). Emitter/absorber interface of CdTe solar Cells. J. Appl. Phys..

[32] Major J.D. (2016). Grain boundaries in CdTe thin film solar cells: A review. Semicond. Sci. Technol..

[33] Amin N., Sopian K., Konagai M. (2007). Numerical modeling of CdS/CdTe and CdS/CdTe/ZnTe solar cells as a function of CdTe thickness. Sol. Energy Mater..

[34] Montoya De Los Santos I., Pérez-Orozco A.A., Liña-Martínez D.A., Courel M., Meza-Avendaño C.A., Borrego-Pérez J.A., Pérez L.M., Laroze D. (2023). Towards a CdTe Solar Cell Efficiency Promotion: The Role of ZnO: Al and CuSCN Nanolayers. Nanomaterials.

[35] Matin M., Amin N., Zaharim A., Sopian K. (2010). A study towards the possibility of ultra thin Cds/CdTe high efficiency solar cells from numerical analysis. WSEAS Trans. Environ. Dev..

[36] Boudour S., Bouchama I., Bouarissa N., Hadjab M. (2019). A study of CdTe solar cells using Ga-doped MgxZn1-xO buffer/TCO layers: Simulation and performance analysis. J. Sci. Adv. Mater. Devices.

[37] Roura Grabulosa P., Vilà i Arbonès A.M., Bosch J., López-de Miguel M., Cornet i Calveras A., Morante i Lleonart J.R., Westwood D.I. (1997). Atomic diffusion induced by stress relaxation in InGaAs/GaAs epitaxial layers. J. Appl. Phys..

[38] Janssen G. (2007). Stress and strain in polycrystalline thin films. Thin Solid Film..

[39] Hegedus S.S., Shafarman W.N. (2004). Thin-film solar cells: Device measurements and analysis. Prog. Photovolt. Res. Appl..

[40] Pokhrel D., Bastola E., Phillips A.B., Heben M.J., Ellingson R.J. (2020). Aspect ratio controlled synthesis of tellurium nanowires for photovoltaic applications. Mater. Adv..

[41] Bhandari K.P., Tan X., Zereshki P., Alfadhili F.K., Phillips A.B., Koirala P., Heben M.J., Collins R.W., Ellingson R.J. (2017). Thin film iron pyrite deposited by hybrid sputtering/co-evaporation as a hole transport layer for sputtered CdS/CdTe solar Cells. Sol. Energy Mater. Sol. Cells.

[42] Marsillac S., Parikh V., Compaan A. (2007). Ultra-thin bifacial CdTe solar cell. Sol. Energy Mater. Sol. Cells.

